# Chirality Transfer in Gold Nanoclusters: Insights from Chiral Spectroscopy and Theoretical Modeling

**DOI:** 10.1021/acsphyschemau.6c00014

**Published:** 2026-04-13

**Authors:** Rareş Banu, Chiara Morassut, Ariel Perez Mellor, Thomas Bürgi, Mauro Stener, Noelia Barrabés

**Affiliations:** † Institute of Materials Chemistry, 27259 TU Wien, Getreidemarkt 9/E165, 1060 Vienna, Austria; ‡ Department of Chemical and Pharmaceutical Sciences, 9315 University of Trieste, Via Giorgieri 1, 34127 Trieste, Italy; § Department of Physical Chemistry, 27212 University of Geneva, 30 Quai Ernest Ansermet, CH-1211 Genéve 4, Switzerland; ∥ Department of Chemical and Pharmaceutical Sciences, University of Trieste, Via Giorgieri 1, 34127 Trieste, Italy

**Keywords:** gold nanoclusters, chirality, spectroscopy, vibrational circular dichroism, density functional calculations

## Abstract

Chirality in atomically precise gold nanoclusters emerges from the interplay among the metal core, the metal–ligand interface, and the ligand shell, yet how these hierarchical elements influence ligand conformation remains unclear. This work investigates chirality transfer from the nanocluster framework to the ligand sphere by using vibrational circular dichroism (VCD) spectroscopy combined with density functional theory and SCC-DFTB calculations. Three enantiopure thiolate-protected gold nanoclusters—Au_25_(2-MeBuS)_18_, Au_38_(2-MeBuS)_24_, and Au_144_(2-MeBuS)_60_—were selected as model systems, representing one, two, and three hierarchical levels of chirality, respectively. VCD analysis reveals a progressive restriction and reorganization of the ligand conformations with increasing structural chirality. While the intrinsically achiral Au_25_ induces only modest spectral changes, the chiral arrangement of the staple units in the Au_38_ induces significant changes in both IR and VCD spectra. In Au_144_, the additional chiral gold core leads to a strong amplification of the VCD response. Computational analysis shows that ligand conformations are governed by cluster geometry, with binding favoring the most stable free ligand conformer. These results provide direct insight into chirality transfer mechanisms in gold nanoclusters, leading to a better understanding of the design of chiral nanomaterials.

## Graphical abstract

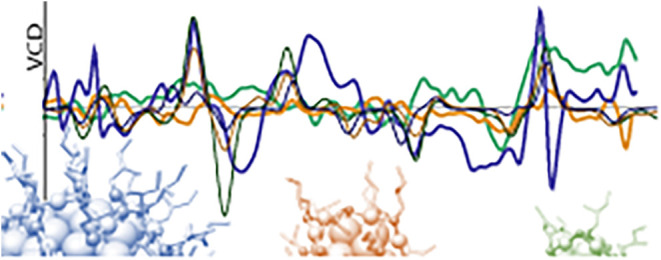

## Introduction

Chirality is a fundamental phenomenon that exists at all levels of matter, from molecules to macroscopic structures. In the rapidly expanding field of nanoscience, an understanding of chirality in nanomaterials has emerged as a key area of interest, as chiral features at this scale can dramatically impact optical, electronic, and biological properties. This enables breakthroughs in areas such as spintronics, photonics, molecular recognition, and biomedicine. However, the unique behaviors and functionalities of chiral nanomaterials often depend on their atomic-level arrangement. This means that detailed investigation and control of chirality with atomistic precision are necessary to fully exploit these effects for advanced device design and targeted applications.^1,2^

Monolayer-protected metal nanoclusters represent a class of atomically precise chiral nanomaterials that bridge the molecular and metallic domains through their well-defined core and ligand architectures. Unlike nanoparticles, nanoclusters exhibit discrete electronic states and atomic arrangements that enable the direct observation and manipulation of chirality at an atomic level. Their metal core, typically gold or silver, is stabilized by a monolayer of ligands (most commonly thiolates, but also phosphines, carbenes, and others), enabling exceptional stability and diverse pathways for chirality.^3–5^

Chirality in gold nanoclusters can be categorized as intrinsic, arising from the atomic organization of the gold core or Au–S interface, or induced, introduced by chiral ligands or postsynthetic transformations.^6^ Intrinsic chirality was first observed in crystal structures of clusters such as Au_38_(SR)_24_,^7^ Au_102_(SR)_44_,^8^ and Au_144_(SR)_60_,^9^ all protected by achiral thiolates, showing that even the metal–ligand interface and ligand arrangement can break mirror symmetry. Induced chirality was demonstrated by using chiral ligands like glutathione, leading to pronounced chiroptical activity and even enantiomerically pure cluster samples.^10,11^ Building on this, Pelayo et al.^12^ provided a comprehensive framework for understanding chirality in bare and ligand-protected metal nanoclusters, showing that for many thiolate-protected gold systems, the dominant contribution to overall chirality arises from the ligand shell and Au–S interface, and introducing quantitative descriptors such as the Hausdorff chirality measure to correlate geometric chirality with chiroptical response.^12,13^

The metal–ligand interface, in particular, the arrangement of staple motifs such as −SR–Au–SR– and −SR–Au–SR–Au–SR–, is often observed to adopt a chiral orientation around an otherwise symmetric core, and this is further modulated by ligand conformational effects and packing. Dynamic aspects of nanocluster chirality have been highlighted, for example, by studies where structurally achiral nanoclusters display chiral responses arising from ligand arrangements or where heteroligand incorporation induces chirality by restructuring staple motifs. Recent work has also uncovered nanoclusters whose intrinsic chirality resides predominantly in the surface layer: Liu and co-workers reported an Au138­(SR)­48 cluster in which highly dynamic aromatic ligands assemble into chiral patterns, imparting an “outside-in” influence while the interfacial −S–Au–S– motifs and metal kernel remain essentially achiral. This demonstrates that surface-layer organization alone can generate intrinsic chirality and expands the spectrum of hierarchical chiral motifs accessible in atomically precise gold nanoclusters.^14,15^

In parallel, new synthetic strategies are being developed to access enantiopure metal nanoclusters in a controlled manner. Asymmetric transformation has emerged as a powerful approach where achiral clusters are converted into chiral products under the influence of chiral auxiliaries. Liu and co-workers recently achieved the asymmetric transformation of achiral Au_23_(SR)_16_ nanoclusters to give enantiomerically enriched products, revealing a negative nonlinear relationship between chiroptical activity and inducer enantiomeric excess, which indicates the involvement of multiple chiral auxiliaries in the transformation pathway and underscores the complexity of chirality induction at the nanoscale.^15^ Together with earlier mechanistic models such as intrinsically chiral cores, dissymmetric field effects, and chiral footprint concepts, these studies emphasize that chirality in nanoclusters can arise and evolve through several coupled structural levels, from the ligand periphery to the metal kernel.

In a recent theoretical study, Häkkinen and co-workers systematically analyzed the different contributions to the chiral response of water-soluble Au_25_(GS)_18_ nanoclusters. They showed that the glutathione ligands populate several conformations and that this conformational ensemble strongly modulates the overall chiroptical response. In addition, they highlighted the critical role of the solvent: explicit water molecules interact with the ligand shell to form a structured solvation layer, which in turn alters the calculated ECD spectrum. Considering ligand conformational flexibility and solvent effects simultaneously was essential to reproduce the experimental data with quantitative accuracy, underlining the need for a comprehensive treatment of all hierarchical elements that contribute to nanocluster chirality.^11^

Building on these advances, our previous work demonstrated that the use of the chiral thiol 2-methylbutanethiol (2-MeBuSH) directs the formation of enantiopure Au_25_, Au_38_, and Au_44_ nanoclusters, each exhibiting a distinct combination of structural elements contributing to overall chirality.^16^ In particular, Au_25_ is intrinsically achiral and derives chirality solely from the ligand, Au_38_ possesses an additional chiral Au–S interface, and Au_44_ combines a chiral core, chiral staple motifs, and chiral ligands, resulting in pronounced “super” chirality in electronic circular dichroism (ECD). These systems thus offer an ideal platform to investigate how chirality is transferred and amplified across different structural levels in a controlled, hierarchical manner.

Vibrational circular dichroism (VCD), complemented by electronic structure calculations, has emerged as a powerful tool to probe chirality transfer and structural effects in such systems. VCD provides conformation-sensitive, chirality-dependent vibrational spectra that report on the handedness and arrangement of surface-bound ligands, while theoretical simulations enable the assignment of VCD features to specific vibrational modes and conformers and clarify how a chiral metal framework can imprint its handedness onto achiral ligands. Early VCD studies on water-soluble nanoclusters have shown that reliable interpretation requires careful consideration of ligand conformations and solvent effects, and that chiral metal clusters can transmit their chirality to adsorbates, leading to distinct VCD signatures sensitive to ligand packing and surface interactions.^17^

Previous work by some of us revealed that achiral thiolates adsorbed on chiral gold clusters adopt chiral conformations, evidenced by distinct VCD signals sensitive to ligand packing and surface interactions.^18^ In yet another account, the possibility of enhancing the VCD response of water-soluble Au_25_ nanoclusters by adding Co­(II) has been demonstrated. The cobalt ions seem to form an adduct with the cluster, by interacting with the ligand shell.^19^

In the present study, we use the enantiopure Au_25_(2-MeBuS)_18_, Au_38_(2-MeBuS)_24_, and Au_144_(2-MeBuS)_60_ nanoclusters as model systems to systematically dissect chirality transfer from the nanocluster framework to the ligand sphere. By combining VCD spectroscopy with density functional theory (DFT) and self-consistent charge density functional tight-binding (SCC-DFTB) calculations, we investigate how the interplay among hierarchical chirality (ligand, interface, and core), staple-motif architecture, and ligand conformational freedom governs the chiral organization of the ligand shell. Focusing on the 1200–1500 cm^–1^ region dominated by ligand bending modes, we show that increasing structural chirality and progressively restricted conformational space lead to reorganized ligand conformations and amplified VCD responses, providing mechanistic insight into chirality transfer and offering design principles for chiral nanomaterials that complement the broader picture established by ECD and geometric chirality analyses.

## Methods

### Experimental Section

The chiral (S)-2-MeBuSH ligand, as well as the Au_25_, Au_38_, and Au_44_ nanoclusters, were synthesized according to previous works in the group.^16^ VCD measurements were done in deuterated DCM, over the course of 8 h per measurement. Details on all procedures are provided in the Supporting Information.

### Computational Model

The IR and VCD experimental spectra of the free ligand are compared with state-of-the-art DFT calculations (TZP basis set and B3LYP functional) in order to obtain a robust and reliable assignment. We are limited to only a harmonic description of normal modes, since it has already been shown for thiols that the only effect of anharmonicity is to shift the peak positions to lower wavenumbers.^20^ Such a shift can be pragmatically implemented by rescaling the wavenumbers by a factor that depends on the method employed (namely, the functional and basis set). We have reported in the Supporting Information the details regarding the rescaling procedure. Once the high-level DFT analysis was completed, we moved to the approximated self-consistent charge density functional tight-binding (SCC-DFTB) method,^21^ which is less accurate but much less demanding numerically. In fact, while present metal clusters are beyond the applicability of high-level DFT methods, SCC-DFTB can be profitably employed in such large systems with reasonable efforts. Moreover, we employed a recent parametrization, which is very accurate for gold–thiolate compounds.^22^ Also for the SCC-DFTB scaling factors, the literature is available (see the Supporting Information for details). The comparison between SCC-DFTB and DFT or experiment is also very useful for assessing the quality of the SCC-DFTB calculations and, therefore, the expected level of deterioration relative to the experiment. For these systems, once the analysis is completed, we move to the comparison between the experiment and theory for the large gold clusters (Au_25_, Au_38_, and Au_44_). For these systems, only SCC-DFTB calculations have been performed. We profitably employed the free ligand analysis to rationalize the IR and VCD spectra of the clusters. It is worth noting that the spectral interval studied in this work (between 1200 and 1500 cm^‑1^) includes only bending normal modes of the ligands; for this reason, the free ligand is very useful for such an analysis. For this reason, we will restrict the analysis to this region in the following. All the DFT and SCC-DFTB calculations have been performed with the AMS program.^23^

## Results and Discussion

Figure 1 shows the three enantiopure thiolate-protected gold nanoclusters investigated in this study, Au_25_(2-MeBuS)_18_, Au_38_(2-MeBuS)_24_, and Au_144_(2-MeBuS)_60_, which were synthesized via a direct protocol based on our previously reported work,^16^ and chosen to represent increasing hierarchical chirality. These atomically precise structures illustrate clusters where chirality originates solely from the ligand (Au_25_), from the ligand plus a chiral Au–S staple interface (Au_38_), and from the combination of a chiral core, interface, and ligand shell (Au_44_), consistent with earlier reports on intrinsically chiral Au_38_ and Au_44_ clusters. Previously, this enabled the observation of a systematic evolution of electronic circular dichroism (ECD) signatures as the number of chiral elements increases. In the present work, vibrational circular dichroism (VCD) spectroscopy is employed to extend this concept and to probe how these distinct structural motifs, individually and in concert, govern the transfer and amplification of chirality within the ligand sphere.

**Figure 1:**
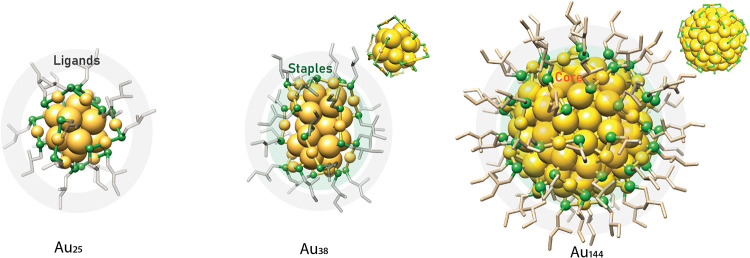
Visual representation of the different chiral levels in the nanoclusters used in this study.

VCD spectroscopy is highly sensitive to conformational changes, making it a powerful probe of how vibrational and chiral information is distributed within flexible ligand shells. Given the structural complexity of thiolate-protected gold nanoclusters in solution, a large ensemble of ligand conformers is expected, and the measured VCD response reflects a population-weighted superposition of their individual contributions. The free 2-MeBuSH ligand exhibits the largest conformational freedom, which becomes progressively restricted upon coordination to the cluster surface. This restriction is further modulated by the nature of the surface motifs: Au_25_ carries only long (dimeric) staples, Au_44_ only short (monomeric) staples, and Au_38_ a mixture of both, so that the available conformational space and steric constraints differ across the three systems. In addition, hierarchical levels of chirality, as well as their transfer from one level to another, are expected to influence the stability and organization of the ligand shell; consistent with previous work on the ECD properties of these structures.^16,18^ By analogy, a similar multilevel transfer is anticipated in the VCD spectra.

### Free Ligand Analysis

Given the demonstrated sensitivity of ligand conformations to cluster chirality, the free 2-MeBuSH ligand was first investigated to establish a conformational baseline for subsequent cluster analysis.

In Figure 2, the IR and VCD spectra of the free ligand are shown. The IR bands in the 1280–1240 cm^–1^ region arise mainly from C–C skeletal stretching modes, coupled with CH_2_/CH bending and contributions from C–S stretching. The two VCD bands at 1280 and 1245 cm^–1^ correlate with the IR bands of the sample. A weaker VCD response appears with the IR band at 1460 cm^–1^, assigned to CH_2_ scissoring and CH_3_ asymmetric bending, and the small IR shoulder at 1430 cm^–1^, with a corresponding weak VCD band, likely represents a secondary component of the CH_2_ scissoring vibration.

**Figure 2:**
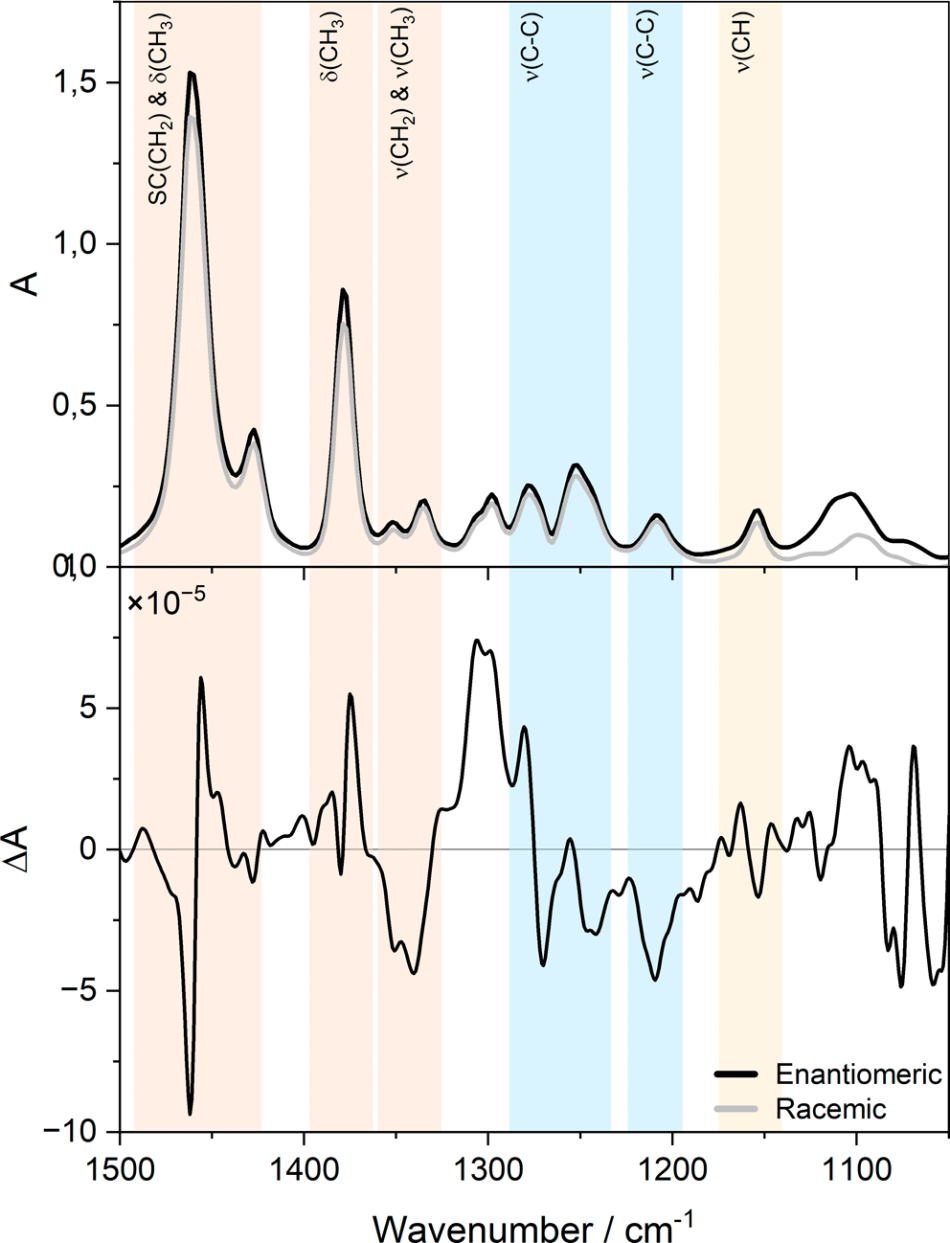
Top panel: IR spectra of the racemic and enantiomeric 2-MeBuSH ligand. Bottom panel: VCD spectrum of the 2-MeBuSH ligand.

A pronounced band at 1375 cm^–1^, with a comparatively high anisotropy factor, is attributed to the symmetric bending (umbrella) mode of CH_3_ groups; because one methyl group is directly attached to the stereogenic center, the strong VCD response is consistent with its proximity to the chiral carbon. The smaller IR bands at 1350–1330 cm^–1^, which exhibit relatively intense VCD signals, likely originate from deformations of the methyl and methylene groups adjacent to the chiral center. In the 1310–1300 cm^–1^ range, two weak IR bands show VCD signals of opposite sign, a pattern plausibly explained by the coexistence of multiple conformers that possess similar electric dipole strengths but opposite magnetic dipole directions. Finally, the IR bands at 1207 and 1100 cm^–1^ are assigned to C–C stretching and CH deformations near the chiral center, in agreement with their strong VCD activity, whereas the band at 1155 cm^–1^ is VCD-inactive, indicating that the corresponding vibrations originate from groups more distant from the stereogenic center.

Building directly on these experimental assignments, the comparison with calculated IR and VCD spectra for the free ligand, considering both the most stable conformer (C2) and a Boltzmann-weighted average over all identified conformers, provides further insight into the conformational landscape (Figure 3a,b). Theoretical wavenumbers are scaled according to literature protocols,^24^ yielding optimal factors of 0.975 (B3LYP-TZP) and 0.99 (SCC-DFTB) by matching the IR profile of C2 to experiment; these are then applied consistently to the VCD spectra. While a full Boltzmann average or molecular dynamics simulation would ideally capture the entire conformational ensemble, the high sensitivity of relative populations to small DFT energy differences complicates this approach. Therefore, analysis begins with C2, which dominates roughly one-third of the population.

**Figure 3:**
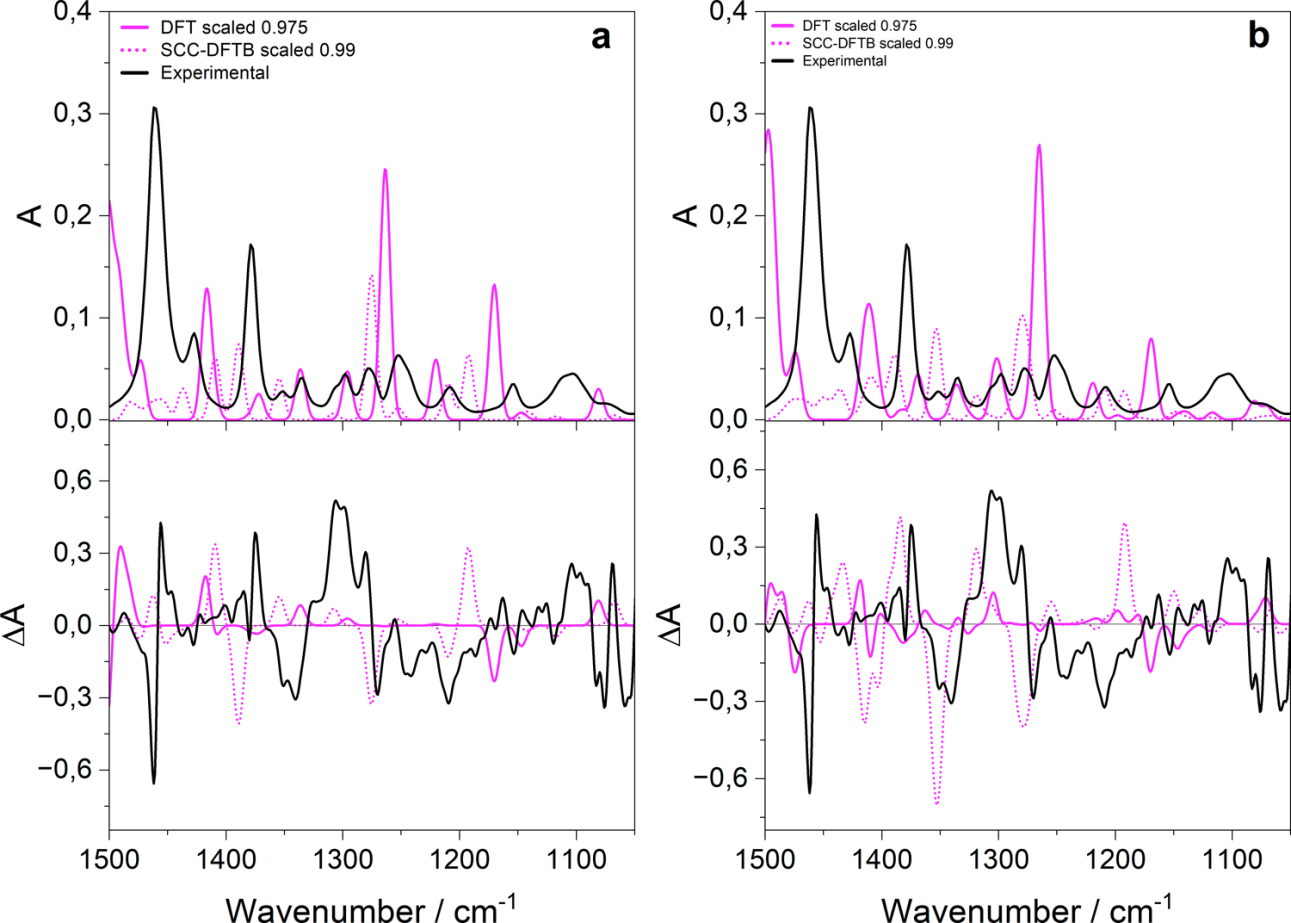
Experimental and calculated (scaled) IR (top panel) and VCD (bottom panel) spectra of the most stable conformer (a) and of the average of conformers (b) of the free 2-MeBuSH ligand. Data scaled along the wavenumber axis according to the literature.^24^

The DFT IR profile matches the experiment well for key features around 1450 cm^–1^ (intense peak and a weaker shoulder), 1375 cm^–1^, and just below 1350 cm^–1^, though the region of 1200 to 1300 cm^–1^ shows discrepancies, including an overestimated peak near 1225 cm^–1^ (absent in the racemate but present in the enantiomer). SCC-DFTB yields similar trends but underestimates the 1450 cm^–1^ intensity and overpredicts a peak at 1261 cm^–1^, as expected from its approximations, though such semiempirical methods often improve for larger systems. For VCD (Figure 3a, bottom), DFT satisfactorily captures the 1450 cm^–1^ feature (especially the negative component) and the positive 1382 cm^–1^ peak, but underestimates signals near 1340 and 1300 cm^–1^ and struggles below 1250 cm^–1^; SCC-DFTB performs worse overall, with a weaker response of 1450 cm^–1^ and shifts in the 1375–1340 cm^–1^ region.

Notably, IR spectra show only minor changes between single-conformer (C2) and averaged calculations, confirming low conformational sensitivity, whereas VCD improves markedly with averaging, a better matching experiment for both methods, and underscoring VCD’s high sensitivity to dipole moment orientations via the Rosenfeld equation. This conformational averaging proves essential for the free ligand but becomes less critical for clusters, where chemical bonding to the Au surface sharply restricts flexibility; indeed, adsorbed ligands closely mirror the free C2 geometry.

To quantify this accurately, the relative root-mean-square deviation (rRMSD) of rotational constants is computed for adsorbed ligands versus free C2 (after graph-based fragmentation; see SI Section 4 and refs 25,26 ), clearly distinguishing C2 from other free conformers. The closest conformer is C10, with an rRMSD = 0.14. C10 differs from C2 by rotation about the dihedral angle linking the *C*_α_ carbon to the ethyl substituent (Figure 4). Across all clusters, bound ligands adopt C2-like backbones with cluster-specific distortions: maximal for isolated ligands on Au_25_ and Au_38_ (RMSD ≈ 0.1, nondihedral in origin), but homogeneously in Au_44_, consistent with short staples enforcing tighter packing.

**Figure 4:**
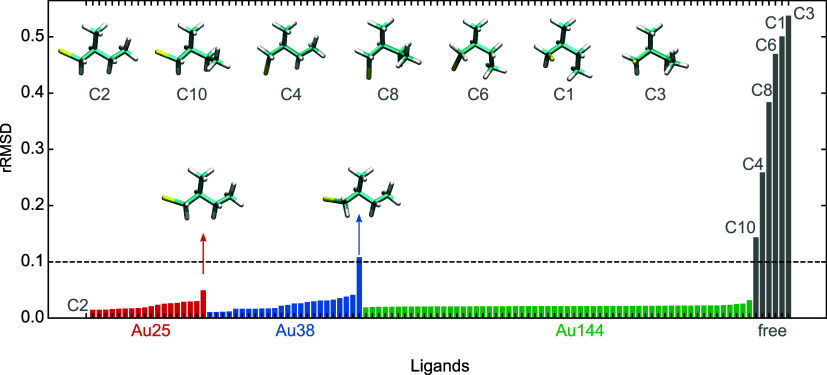
Relative RMSD of rotational constants with respect to the C2 conformation. Each bar corresponds to one ligand geometry, and bars are color-coded by the parent cluster from which the ligand was extracted (Au_25_: red; Au_38_: blue; Au_44_: green), while gray bars denote the set of isolated (free) optimized conformers (C_*i*_ labels). Representative conformers are shown as molecular renderings above the plot (including the C2 reference), and two illustrative cases are highlighted by arrows to emphasize ligands that deviate more strongly from the reference within the Au25 and Au38 subsets.

Normal mode analysis further confirms the vibrational character of key bands and the consistency between the DFT and SCC-DFTB descriptions (Table 1). All modes in the 1200–1500 cm^–1^ region correspond to CH bending vibrations, with the strongest IR intensities for scissoring (A, B) and wagging (D, H) modes, as visualized in Figure 5. Wavenumbers are scaled as previously described, and while full IR/VCD intensities are reported for DFT, only wavenumbers are available from SCC-DFTB, which nevertheless closely tracks DFT positions.

**Figure 5:**
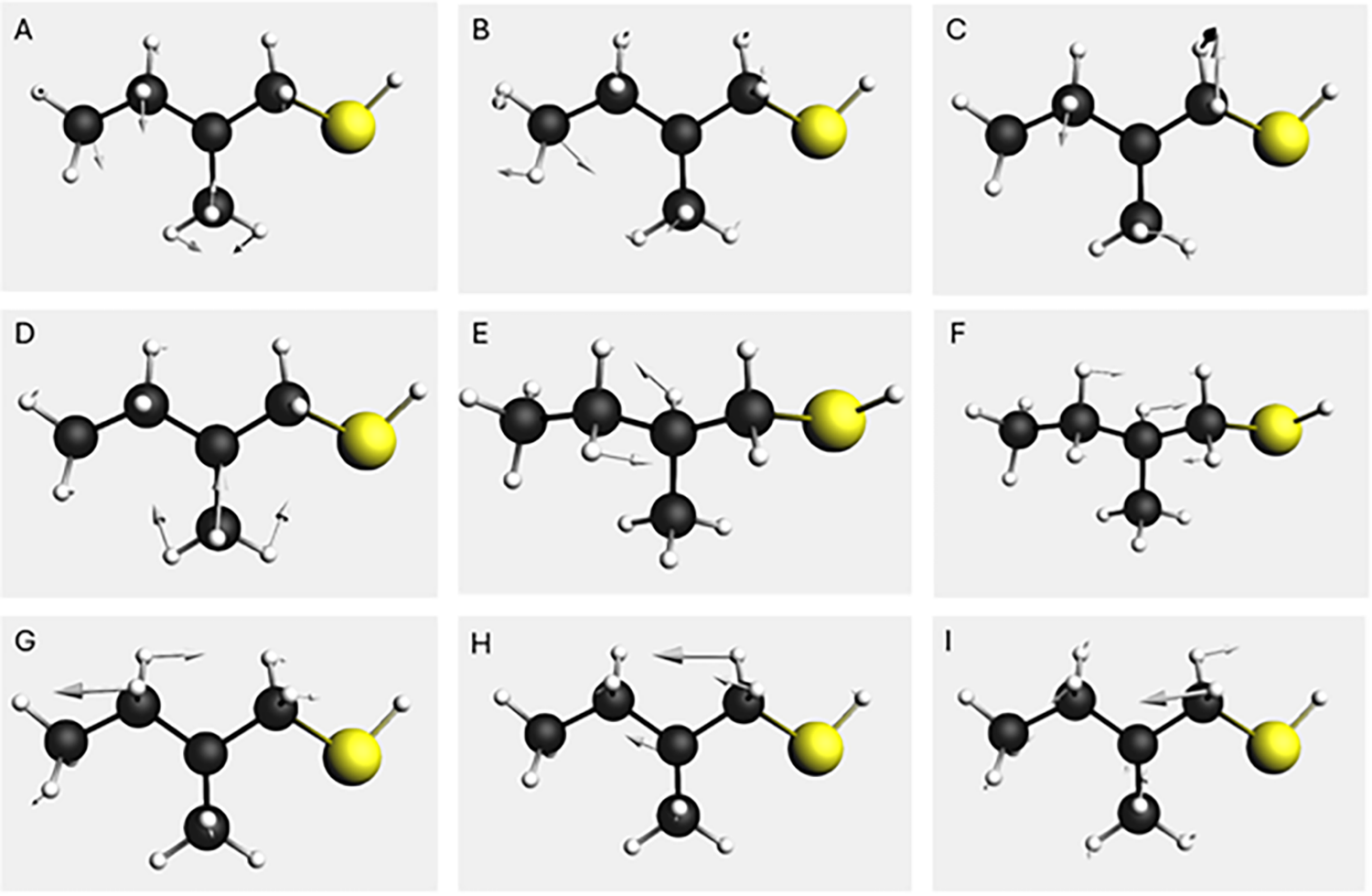
Normal modes of the free ligand calculated by DFT in the range 1200 – 1500 cm^–1^, visualized using the AMS graphical interface.

**Table 1:** List of the Normal Modes of the Free Ligand in the Range 1200–1500 cm^–1^ (Scaled)^a^

feature	DFT wavenumber	DFT IR int.	DFT VCD int.	SCC-DFTB wavenumber
A	1463	12.33	–33.3	1468
B	1454	8.10	31.3	1438
C	1436	3.97	–0.9	1423
D	1382	8.42	24.0	1395
E	1338	1.74	–3.0	1375
F	1303	3.49	8.9	1340
G	1264	3.34	3.1	1261
H	1232	17.82	–0.3	1240
I	1190	4.15	0.6	1198

a Wavenumber (cm^–1^), IR intensity km·mol^–1^, and VCD intensity 10^–44^·esu^2^·cm^2^. For SCC-DFTB, only wavenumbers are reported.

#### Cluster Spectral Evolution

Once the free ligand was evaluated, each cluster was studied, complementing the experimental VCD with DFTB calculations.

Across Au_25_, Au_38_, and Au_44_, the IR spectra evolve slightly from the free ligand reference, while VCD reveals dramatic changes that seem to scale with hierarchical chirality levels (Figure 6).

**Figure 6:**
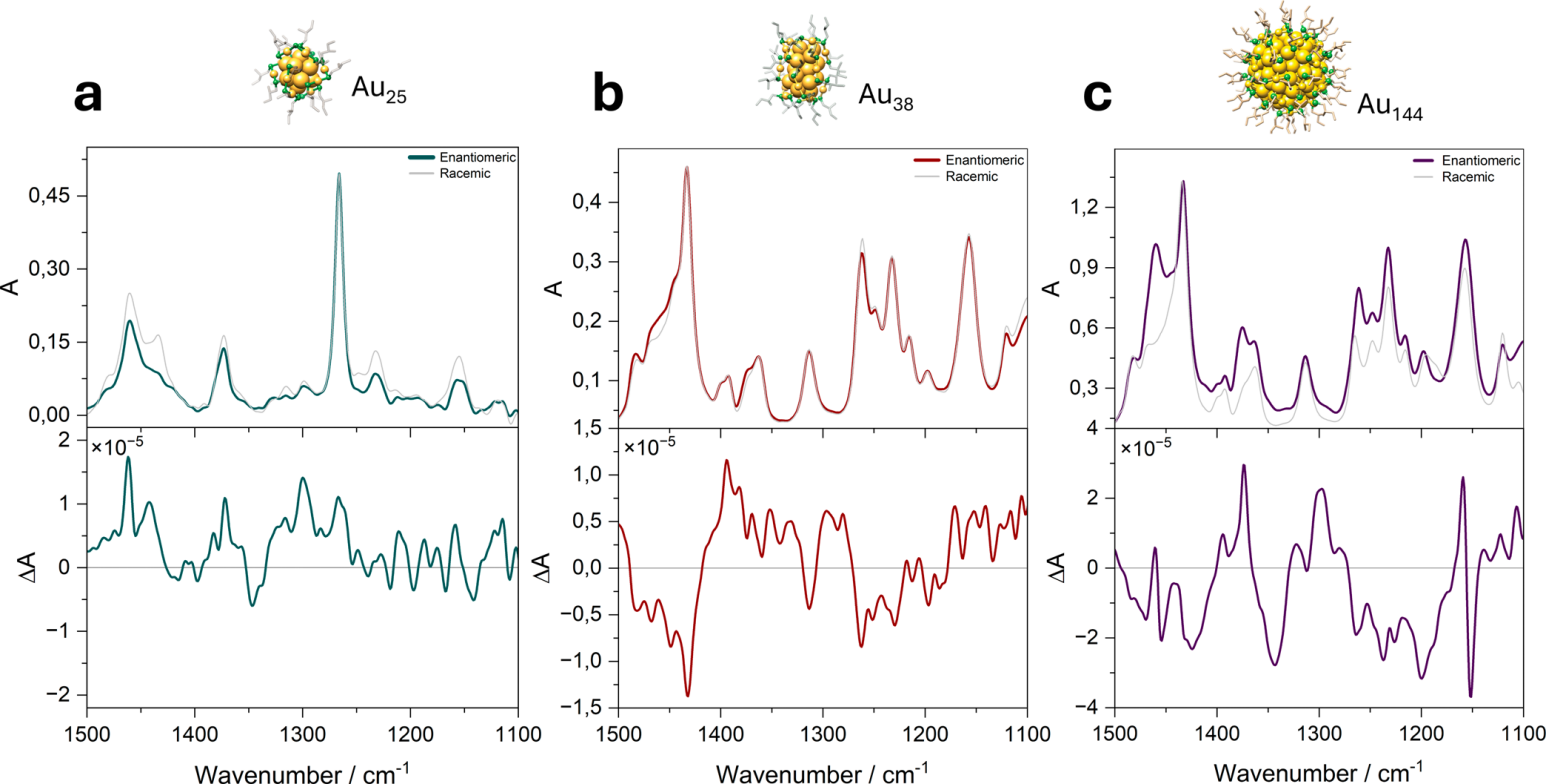
Top panels: IR spectra of the racemic and enantiomeric Au_25_(2-MeBuS)_18_, Au_38_(2-MeBuS)_24_, and Au_144_(2-MeBuS)_60_ nanoclusters. Bottom panels: VCD spectra of the three nanoclusters.

For the achiral Au_25_ (long staples only), coordination introduces new IR bands at 1490/1480 cm^–1^ (shifted from free ligand 1460/1430 cm^–1^) with strong VCD activity, a sign swap at ∼1295 cm^–1^, loss of splitting at 1260 cm^–1^, and a new 1230 cm^–1^ feature near the C–S site; distal CH_3_ umbrella (1375 cm^–1^) and backbone modes (1350–1330 cm^–1^) are largely conserved. SCC-DFTB captures the 1400–1350 cm^–1^ region and 1250 cm^–1^ shape well but underestimates the intensity of 1450 cm^–1^ and overpredicts some low-wavenumber peaks (Figure 7). The VCD agreement is qualitative with the central 1400–1325 cm^–1^ region being too negative.

**Figure 7:**
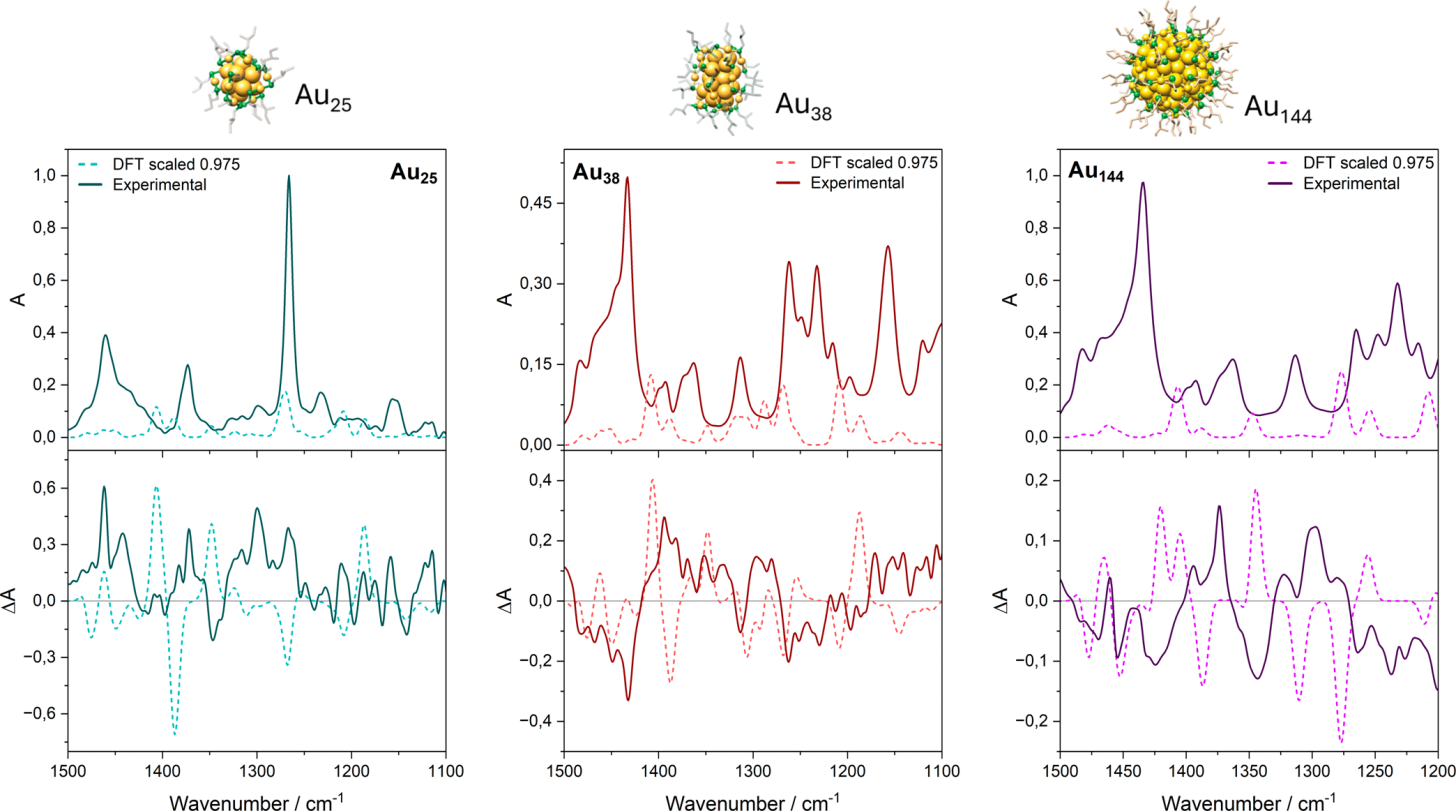
Experiment and calculated IR (top panels) and VCD (bottom panels) of Au_25_, Au_38_, and Au_44_.

In the achiral Au_25_, ligands are coordinated through dimeric (long) staple units only, rendering them less flexible than in free solution; however, with the core intrinsically achiral, this restriction remains modest; therefore, changes in IR and VCD upon coordination are expected to be relatively small. In the higher-wavenumber region (Figure 6a), new IR bands emerge at 1490/1480 cm^–1^—both VCD-active, the latter with a high anisotropy factor. Likely this feature corresponds to the free ligand 1460 cm^–1^ band and its 1430 cm^–1^ shoulder (shifted to 1440 cm^–1^ with enhanced magnetic dipole), plus new VCD activity at 1425–1410 cm^–1^. Distal CH_3_ umbrella (1375 cm^–1^) and backbone modes (1350–1330 cm^–1^) are positionally conserved (slight VCD shifts for the latter, as expected), while the 1295 cm^–1^ VCD band flips sign (conformational hint), 1260 cm^–1^ retains negative in VCD but loses racemate splitting (sharper response), a new 1230 cm^–1^ C–S-proximal feature appears, and 1200/1150 cm^–1^ (CH/C–C near chiral center) show strong VCD.

SCC-DFTB (Figure 7) captures 1400–1350 cm^–1^ and 1250 cm^–1^ shapes well but underestimates 1450 cm^–1^ intensity, overpredicts some low-wavenumber peaks, and yields only qualitative VCD agreement—with the central 1400–1325 cm^–1^ region too negative

The chiral Au_38_ interface (mixed long/short staples forming helical patterns at the bi-icosahedral core ends) drives profound spectral reorganization versus the free ligand and achiral Au_25_, consistent with prior evidence of interlevel chirality transfer (Figure 6b). In the high-wavenumber region, the 1480 cm^–1^ band persists but exhibits VCD sign inversion, the prominent 1460 cm^–1^ peak (strong in free ligand/Au_25_) weakens to a shoulder with inverted VCD, and the weak free ligand 1430 cm^–1^ shoulder strengthens markedly with positive VCD.

Lower-wavenumber changes are equally striking: new bands emerge at 1390 cm^–1^ (weak VCD, likely CH_2_ deformation), 1360 cm^–1^ (strong positive VCD, suggesting multiple CH_3_ umbrella modes from mixed-staple-induced conformations), and—most diagnostic—1310 cm^–1^ (intensely VCD-active, unique to Au_38_ and absent in Au_25_, directly evidencing chiral ligand packing at the interface). The 1370 cm^–1^ CH_3_ umbrella flips to negative VCD (high anisotropy, opposite to free ligand/Au_25_), while 1260/1230 cm^–1^ bands intensify with sign inversion and 1200 cm^–1^ weakens; 1150 cm^–1^ persists but less prominently. Collectively, systematic VCD inversions, emergent bands, and intensity redistributions could be related to restricted conformational access due to the transfer of chirality from the chiral interface to the ligands, thereby reducing the number of accessible conformers and imposing spatially ordered ligand arrangements imposed by the chiral Au–S framework.

SCC-DFTB calculations for Au_38_ (Figure 7) reproduce the observed spectral reorganization with higher accuracy, as compared to the Au_25_. Peak positions and relative intensities align closely with proper distribution across the spectrum; the sole systematic discrepancy is a 25 cm^–1^ shift of the dominant 1425 cm^–1^ feature to 1400 cm^–1^, with smaller blueshifts for subsequent peaks.

Notably, strong experimental bands now emerge between 1200 and 1250 cm^–1^, precisely where theory predicts them—features previously computed but experimentally elusive for both the free ligand and Au_25_. Similarly, the computed VCD profile in the critical 1400–1325 cm^–1^ region matches experiment well upon applying the same minor shift required for IR, confirming the method’s enhanced reliability for this intermediate-sized system with greater electronic delocalization, short staple units, and chiral interface.

Au_44_, which combines a chiral gold core with a chiral Au–S interface built exclusively from short staples, introduces a third hierarchical level of chirality and leads to a pronounced amplification of the VCD response. At the same time, its IR spectrum closely resembles that of Au_38_, indicating that the Au–S interface continues to govern the local ligand geometries (Figure 6c), while the chiral core primarily enhances long-range chiral coupling within the ligand shell. The exclusive presence of short staples brings the ligands into closer proximity, increasing steric crowding and reducing conformational freedom, which is reflected in the narrow and homogeneous rRMSD distribution obtained for Au_44_ ligands.

High-wavenumber patterns in Au_44_ partially revert toward those seen in achiral Au_25_ and the free ligand, for example, the VCD signals at 1480 and 1460 cm^–1^ both become positive (as in Au_25_/free ligand) and the 1430 cm^–1^ band grows strong again—but key markers of the chiral Au_38_ interface continue to evolve further: the 1390 cm^–1^ VCD signal flips sign relative to Au_38_, the 1370 cm^–1^ CH_3_ umbrella mode returns to positive VCD (matching Au_25_/free ligand, opposite to Au_38_), and the diagnostic 1310 cm^–1^ interface band (absent in Au_25_) intensifies substantially. At lower wavenumbers, the chiral core’s influence becomes even clearer through modulated coupling: the 1260 cm^–1^ band realigns with Au_25_ ’s sign (reversing Au_38_’s inversion), 1230 cm^–1^ remains negative (Au_38_ like), 1200 cm^–1^ reemerges strongly (Au_25_ like, suppressed in Au_38_), and 1150 cm^–1^ develops a characteristic bisignate (positive–negative) VCD couplet arising from coupled skeletal stretching and CH bending modes across the dense ligand shell.

SCC-DFTB calculations for Au_44_ (Figure 7) provide the strongest agreement yet, further surpassing Au_38_ performance and confirming the model’s steadily improving accuracy with increasing cluster size and ligand confinement through shorter staples and added levels of chirality. The 1200–1300 cm^–1^ region matches experiment quantitatively, while wavenumbers from 1300 to 1500 cm^–1^ exhibit a systematic 50 cm^–1^ redshift in both IR and VCD spectra, discrepancies that are consistently smaller than those observed for smaller systems.

So far, we have found that the agreement between theory and experiment improves as the cluster size increases and the staple shortens. This phenomenon has been previously observed in reports, which showed how approximate methods improve with the system size.^27^ Another possible contribution to the increase in accuracy could be the changing staple interface with an increasing cluster size. As the size increases, the staple shortens, and this tends to block or at least strongly reduce the ligand conformational freedom. In this case, the assumption that only the most stable configuration is considered becomes more realistic as the cluster size increases, which explains the observed trend in agreement between theory and experiment. This explanation is corroborated by comparing the calculated VCDs for the series of gold clusters in Figure 8: the profiles show a smooth evolution, indicating that only one conformation is present in the calculated model. In the experiment, the trend is irregular due to the conformational freedom. These combined experimental and computational results reveal how three hierarchical levels of chirality (ligand → Au–S interface → Au core) progressively reorganize ligand conformations and their collective arrangement. Multiple VCD sign inversions relative to Au_38_, most strikingly at 1390, 1370, and 1260 cm^–1^, demonstrate the chiral core’s ability to modulate interfacial chirality transfer, while dramatic signal amplification for Au_44_ underscores nonlinear enhancement from multilevel coupling (Figure 8).

**Figure 8:**
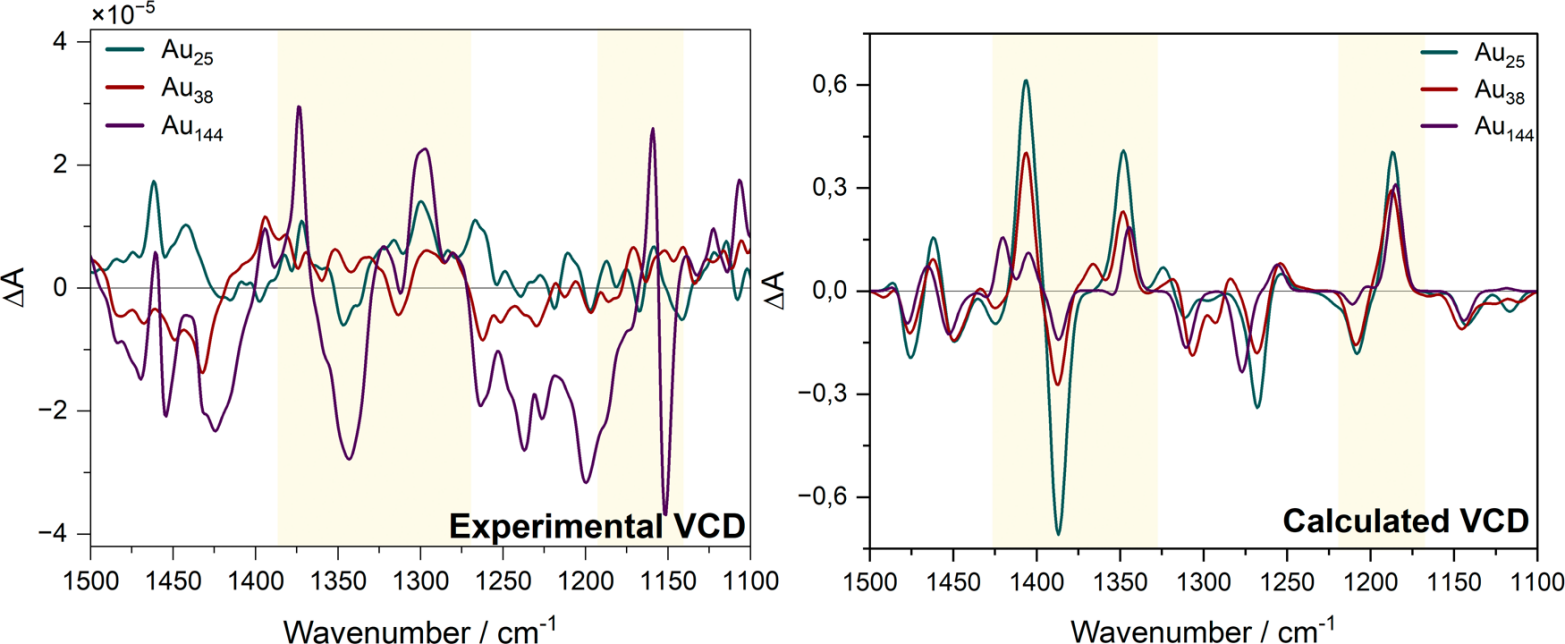
Comparison of the experimental and calculated VCD spectra of the three nanoclusters.

## Conclusions

In conclusion, VCD spectroscopy combined with SCC-DFTB calculations reveals how hierarchical chirality in atomically precise gold nanoclusters propagates conformational bias and spatial organization throughout the ligand shell. The intrinsically achiral Au_25_ core permits modest spectral perturbations from coordination alone, while the chiral Au_38_ interface induces dramatic VCD sign inversions and emergent bands diagnostic of restricted conformers and surface-level chirality transfer; Au_44_’s chiral core then amplifies these effects nonlinearly without altering local IR signatures, highlighting long-range modulation through the densely packed shell (short staples).

Across all cluster sizes, bound ligands preferentially adopt the most stable free ligand backbone conformation, underscoring the dominance of intrinsic conformational stability; nevertheless, cluster-specific interligand coupling gives rise to distinct collective VCD responses.

The systematic improvement in theoretical agreement with an increasing cluster size enables reliable vibrational mode assignments and establishes a foundation for future ensemble-based simulations to resolve shell-level dynamics in greater detail.

Together, these results provide atomistic insight into multilevel chirality transfer and offer general design principles for engineering chiral nanomaterials with a tunable optical activity.

## Supplementary Material



## Notes

Details on the synthesis of all nanoclusters, the ligand, and the characterization, as well as the theoretical calculations (PDF)
