# Supplementary material for: Chirality Transfer in Gold Nanoclusters: Insights from Chiral Spectroscopy and Theoretical Modeling
Source: ACS Phys Chem Au. 2026 Apr 13;6(3):552–60. doi: 10.1021/acsphyschemau.6c00014 (PMC13220208; doi:10.1021/acsphyschemau.6c00014)
Supplement: Supplementary file 1 [file pg6c00014_si_001.pdf]

Supporting Information

Chirality Transfer in Gold Nanoclusters: Insights from Chiral Spectroscopy and Theoretical Modeling

Rareș Banu,[1] Chiara Morassut,[2] Ariel Perez Mellor,[3]
Thomas Bürgi,[3]* Mauro Stener,[2]* Noelia Barrabés[1]*

09/04/2026

[1]Institute of Materials Chemistry, TU Wien, Getreidemarkt 9/E165, 1060 Vienna, Austria
[2]Department of Chemical and Pharmaceutical Sciences, University of Trieste, Via Giorgieri 1, Trieste, Italy
[3]Department of Physical Chemistry, University of Geneva, 30 Quai Ernest Ansermet, CH-1211 Genève 4

1 Chemicals

The reagents, solvents and other consumables used in the preparation of both ligand and nanoclusters were obtained from commercial suppliers. Hydrogen tetrachloroaurate trihydrate, $HAuCl_4 \cdot H_2O$ (49.0 % Au basis) was obtained from Alfa Aesar.(S)-(-)-2-methyl-1-butanol (98.0+ %) was ordered from TCI Chemicals. Methylenechloride D2 (99,80 %) was ordered from Eurisotop. Triethylamine (99 %) was purchased from Acros Organics. 4-Toluenesulfonyl chloride (98 %) and tetraoctylammonium bromide, TOABr (98 %) were ordered from abcr Gute Chemie. Hydrochloric acid, HCl (37 %) was purchased from Carl Roth. Thiourea (for analysis), sodium hydroxide, NaOH (puriss.), dichloromethane, DCM (99.9+ %), and sodium tetrachloropalladate, Na_2PdCl_4 (98 %) were attained from Sigma-Aldrich. Sodiumborohydride, $NaBH_4$ (95+ %) was ordered from Chem Lab NV. Milli-Q water (resistivity of 18.2 MΩ· cm at 25 °C) was used in the synthesis procedure. All solvents used (ethanol, methanol, tetrahydrofuran, toluene, etc.) were at least of synthesis grade and used without further purification. Bio Beads S-X1 support (Bio-Rad) was used for size exclusion chromatography (SEC) and silica gel 60 for column chromatography.

2 Synthesis

2.1 (S)-2-methyl-1-buthanethiol (2-MeBuSH)

The (S)-enantiomer of the chiral thiol ligand used for the spectroscopic measurements and for the nanocluster synthesis was prepared according to previous experience in the group [1]. First, 17.2 mL (160 mmol) of (S)-2-methylbutan-1-ol and 32.73 g (177 mmol) of 4-toluenesulfonyl chloride were dissolved in 60 mL of dichloromethane (DCM). To the ice-cooled mixture, 55.2 mL (395 mmol) of triethylamine were slowly added, forming a white precipitate. The reaction mixture was stirred at room temperature for 16 hours, after which The precipitate was then removed, and the solution was washed with diluted HCl and water. The aqueous phase was extracted with DCM, and the combined organic layers were dried over Na_2SO_4. After evaporating DCM using a rotary evaporator, a clear oil was obtained, which was further purified by silica column chromatography using a 1:3 hexane:EtOAc mixture (EtOAc = ethyl acetate). Next, the purified intermediate (31.3930 g, 130 mmol) was dissolved in 185 mL of ethanol

(EtOH) along with 19.7151 g (259 mmol) of thiourea. The mixture was refluxed at 80 °C for 72 hours, followed by the addition of 60 mL of 20 % NaOH, and heating at 80 °C for another 60 minutes. After cooling to room temperature, the solution was acidified with 100 mL of 10 % HCl. The organic phase was extracted using hexane and dried over Na_2SO_4. Finally, the solvent and impurities were removed by distillation at 140 °C.

2.2 Au_{25}(2-MeBuS)$_{18}$

Au_{25}(2-MeBuS)$_{18}$ nanoclusters, as well as the corresponding cluster with the racemic ligand were synthesized according to previous accounts in the group.[1] To start with, 250 mg (0.64 mmol) $HAuCl_4 \cdot 3\,H_2O$ were mixed with 416 mg (0.76 mmol) tetra-n-octyl-ammonium-bromide (TOABr) and dissolved in 25 mL tetrahydrofuran (THF). After adding 784 µL (S)-2-methyl-1-buthanethiol (2-MeBuSH) (or the racemic version), the solution was stirred for 1 h, until it became clear. Subsequently, 480 mg $NaBH_4$ were dissolved in 10 mL cold nanopure water and rapidly added, resulting in the reduction of the gold. The mixture was stirred for 4 days, after which the organic phase was evaporated and the solid washed with methanol. Finally, the clusters were refined by size exclusion chromatography on Bio-Beads SX-1 in THF.

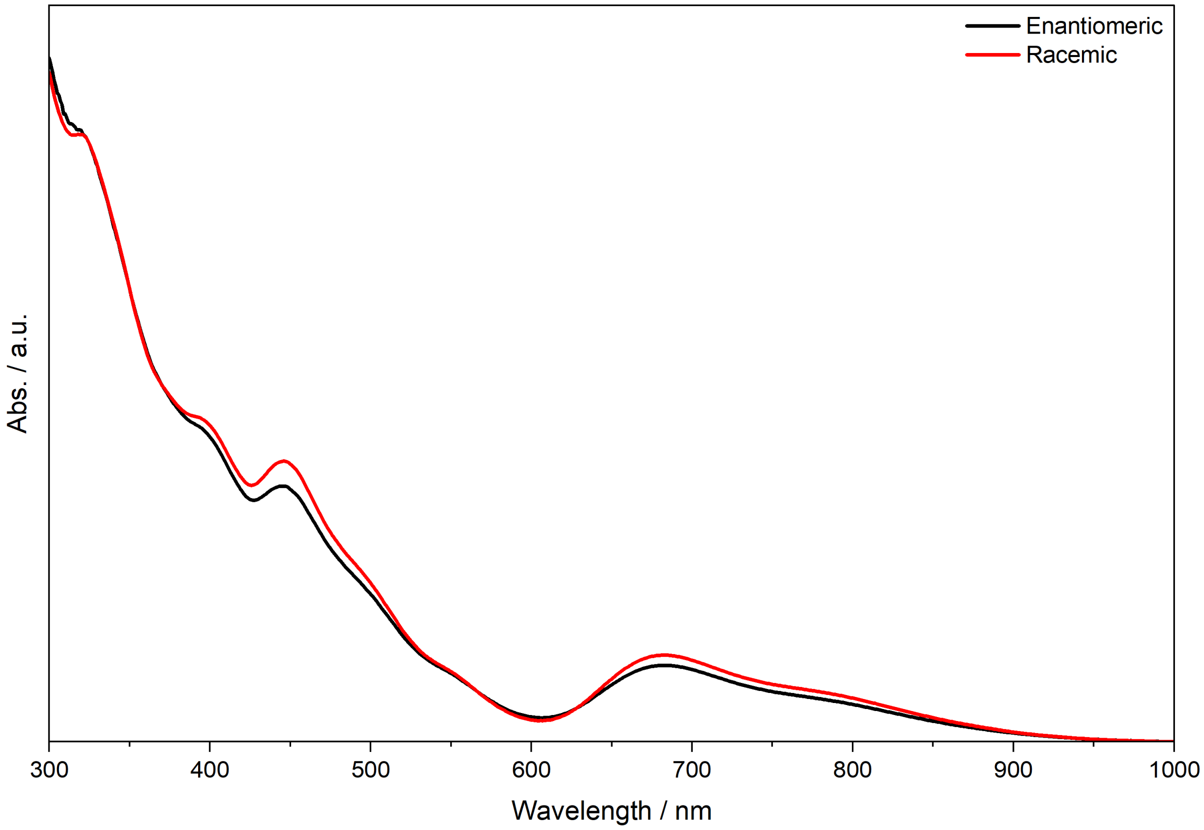

Figure 1: UV-Vis of the Au_{25} nanoclusters.

2.3 Au_{38}(2-MeBuS)$_{24}$

Au_{38}(2-MeBuS)$_{24}$ and its racemic counterpart were synthesized by adapting the protocol reported by Stellwagen and co-workers[2]. 50 mg (0.15 mmol) $HAuCl_4 \cdot 3\,H_2O$ and 155 mg (0.5 mmol) of L-glutathione (GSH) were dissolved in 8 mL MeOH and 3.5 mL H2O, yielding a white suspension. After cooling the mixture to 0 °C, 47 mg (1.2 S2 mmol) $NaBH_4$ suspended in 2.4 mL ice-cold water were added, which resulted in the formation of a black precipitate. The reaction was stirred at 0 °C for 1 h. After separating the black precipitate by centrifugation, the solid was dissolved in 2.4 mL H_2O, 1.5 mL acetone and 2

mL (15.6 mmol) (S)-2-MeBuSH (or the racemic). The reaction was continued at 80 °C for 16 h. The phases were separated and the aqueous phase washed with DCM. The organic phase was dried and the resulting black precipitate washed with ethanol for purification and refined by SEC.

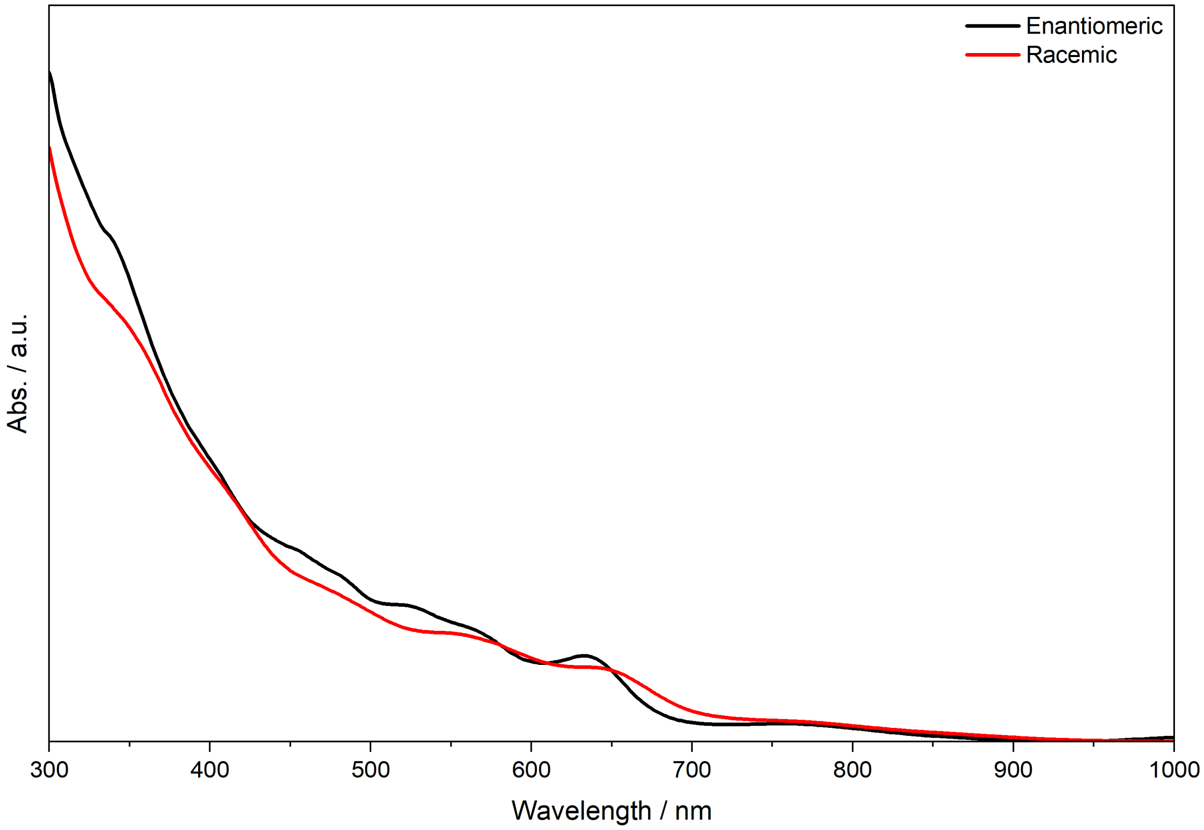

Figure 2: UV-Vis of the Au_{38} nanoclusters.

2.4 Au_{144}(2-MeBuS)$_{60}$

Au_{144}(2-MeBuS)$_{60}$ nanoclusters were prepared by modifying the procedure presented by Qian et al.[3] 236 mg (0.6 mmol) $HAuCl_4 \cdot 3\,H_2O$ were mixed with 380 mg (0.7 mmol) TOAB and dissolved in 30 mL MeOH. The red solution was stirred for 15 minutes at room temperature, after which 394 µL (3.2 mmol) 2-MeBuSH (or the racemic) were added, giving a white suspension. After stirring for 15 min at room temperature, the polymer suspension was reduced using a cooled solution of 227 mg (6 mmol) $NaBH_4$ dissolved in 12 mL water, yielding a black precipitate. The black solution was stirred for another 5 h at room temperature. Subsequently, the black precipitate was separated by centrifugation and washed several times with methanol. The crude product was purified by SEC.

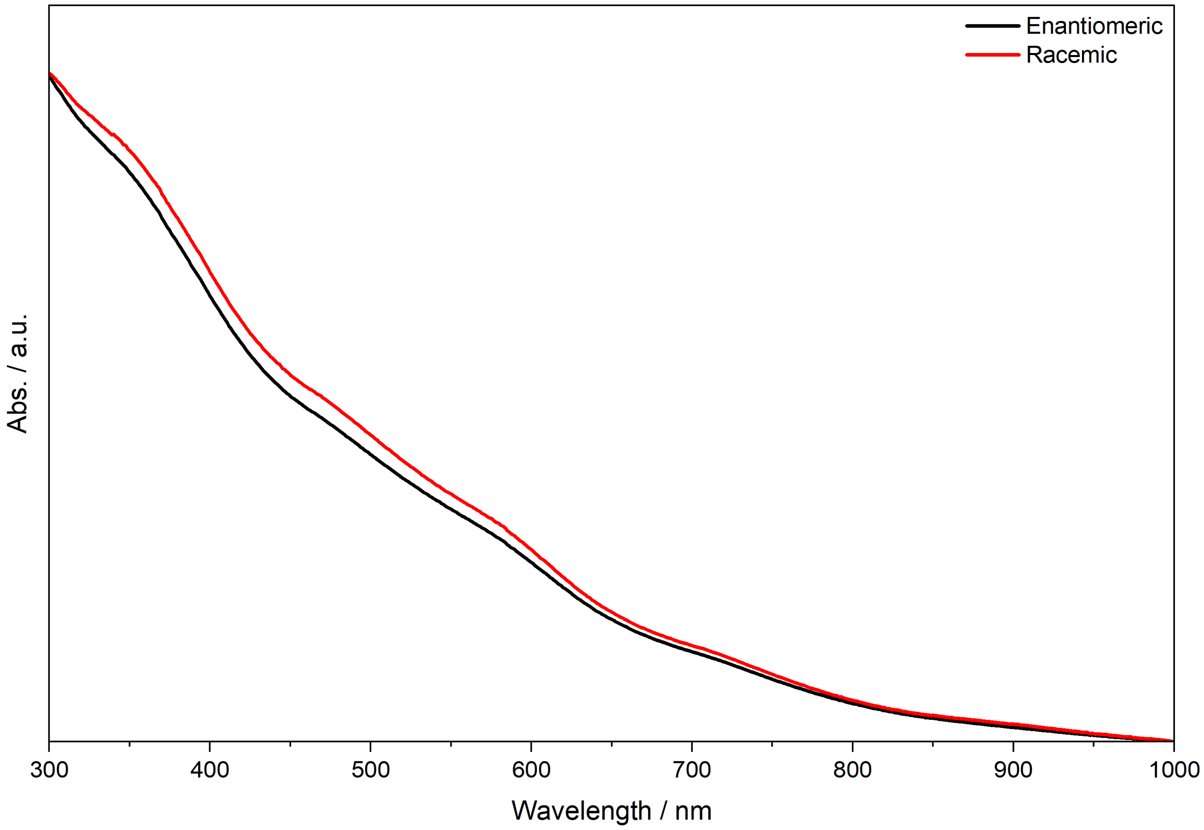

Figure 3: UV-Vis of the Au_{144} nanoclusters.

3 Characterization

3.1 Ultraviolet-Visible Spectrscopy

UV-Vis spectroscopy was performed on a UV-1600PC spectrometer using cuvettes of 1cm pathlength, in DCM.

3.2 Vibrational Circular Dichroism (VCD) Spectroscopy

VCD spectroscopy was performed on a Brucker Vertex 80V IR spectrophotometer, equipped with a PMA-50 module. The spectra were taken in deuterated DCM, with 8 accumulations of 1 hour each, with a resolution of 4 cm^{-1}, in a cuvette with a pathlength of 2 mm. All spectra hve been solvent corrected.

The following graphs show the full spectral range used for the VCD measurements. The signals below 1500 cm^{-1} were disregarded in the VCD, as no IR absorption is present after this point.

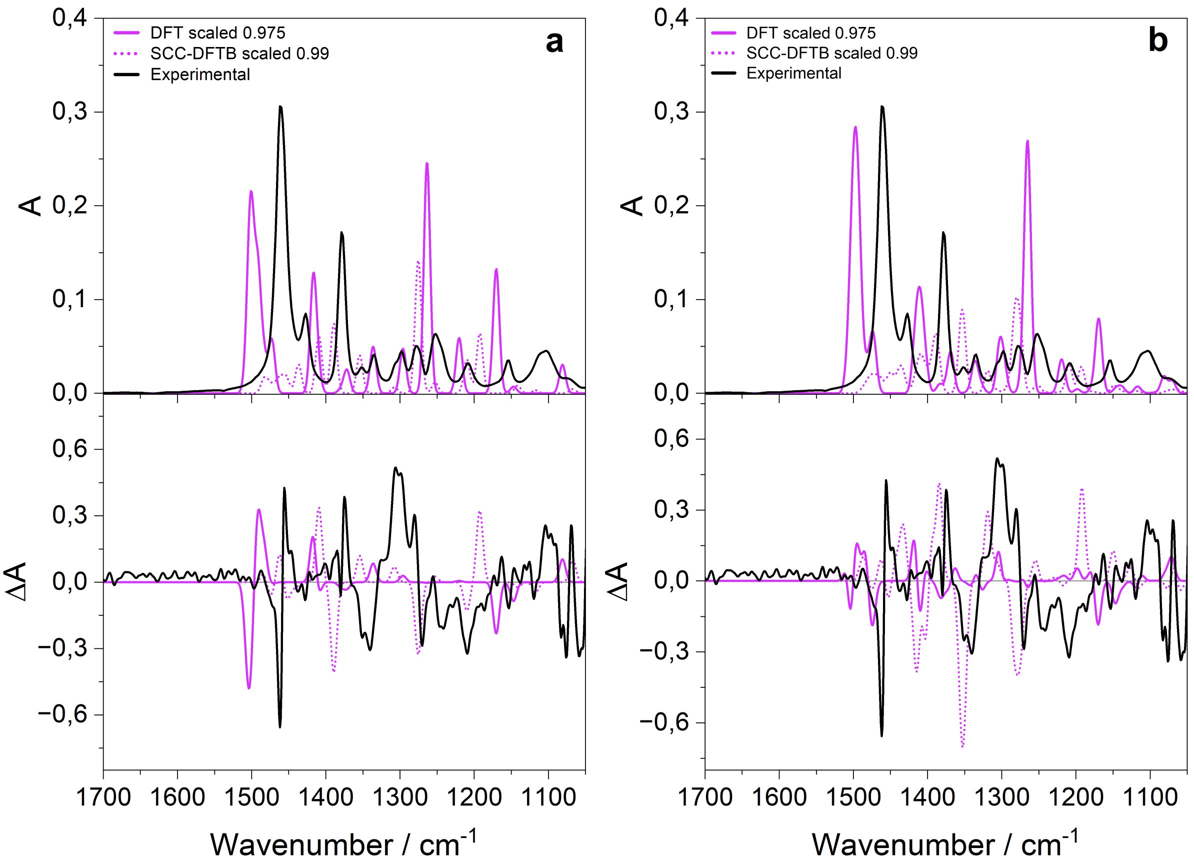

Figure 4: a: C2 conformer, B: average of conformers

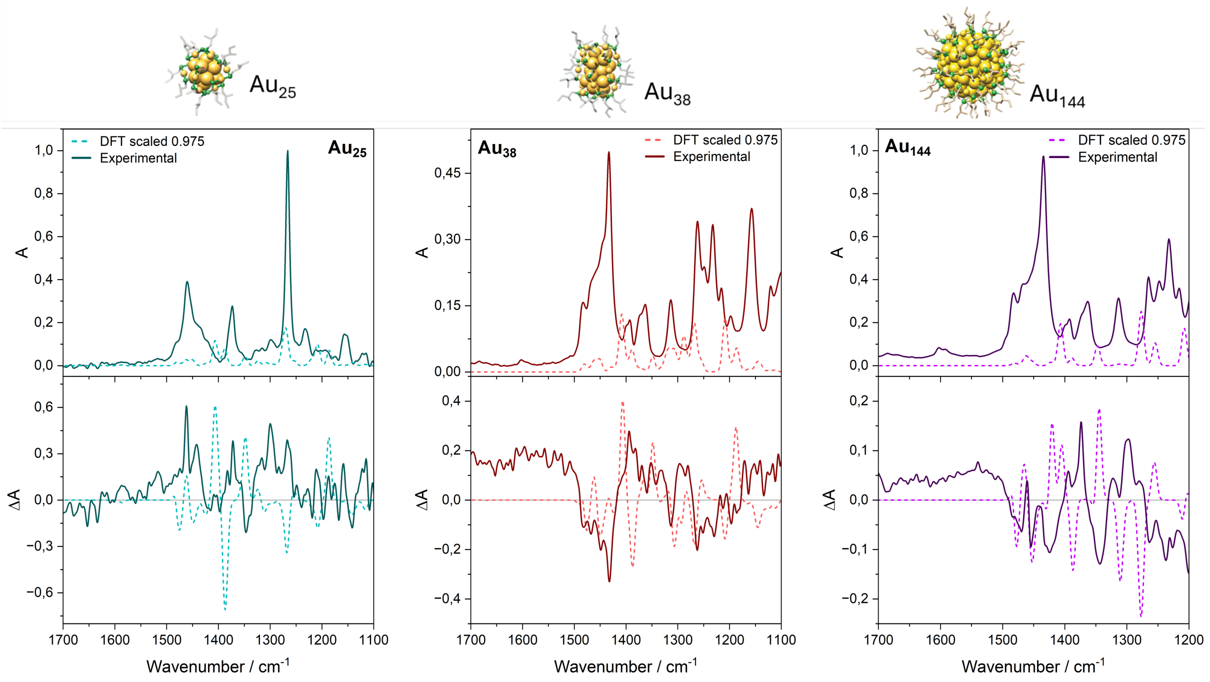

Figure 5:

4 Graph-based extraction and conformational analysis of the ligand shell

Ligand conformations were extracted from the optimized Au–ligand cluster XYZ coordinates using a graph-based fragmentation procedure [4, 5]. Before graph construction, Au atoms were excluded (by element symbol filtering), and the remaining atoms were represented as an undirected molecular graph with atoms as nodes and covalent bonds as edges. Edges were assigned using a distance-based criterion derived from tabulated covalent radii [6] and a dimensionless scaling factor $\alpha = 1.20$. The covalent radii used are summarized in Table 1. Atoms i and j were considered bonded when $d_{ij} \leq \alpha\,(R_i + R_j)$. For each XYZ cluster geometry, the adjacency matrix was built from all pairwise distances, and disconnected subgraphs were identified by a depth-first search algorithm, yielding connected components corresponding to individual ligands. Each component was exported as an independent XYZ file containing the ligand geometry.

Rotational constants were then computed for every extracted ligand geometry to provide an alignment-free descriptor of the mass distribution and overall conformation. For each ligand geometry, the center of mass was evaluated from isotopic atomic masses (see Table 1), and the inertia tensor about the center of mass was constructed in units of amu $\cdot$ Å^2. The symmetric inertia tensor was diagonalized with a Jacobi eigen-solver to obtain the principal moments $I_a \leq I_b \leq I_c$, from which the conventional rigid-rotor rotational constants were evaluated as

$$A = \frac{h}{8\pi^2 I_a}, \qquad B = \frac{h}{8\pi^2 I_b}, \qquad C = \frac{h}{8\pi^2 I_c},$$

and reported in GHz. This workflow was applied identically to ligands extracted from the cluster environment and to the ligands optimized in the isolated ("free") environment, enabling a direct comparison of environment-induced conformational changes.

To quantify conformational similarity relative to a common reference, we defined a relative RMSD in rotational-constant space with respect to a chosen reference conformer (label C2, the most stable ligand geometry in the gas phase). For each structure, fractional deviations were computed as $\Delta A/A_0$, $\Delta B/B_0$, and $\Delta C/C_0$, and combined as

$$\mathrm{rRMSD} = \sqrt{\frac{1}{3}\left[\left(\frac{A - A_0}{A_0}\right)^2 + \left(\frac{B - B_0}{B_0}\right)^2 + \left(\frac{C - C_0}{C_0}\right)^2\right]}.$$

Table 1: Element-specific parameters, covalent radii R and atomic masses M, used in the graph-based ligand extraction and in the rotational-constant analysis.

Element	R / Å	M / u
H	0.31	1.007 825
C	0.76	12.000 000
N	0.71	14.003 074
O	0.66	15.994 915
S	1.05	31.972 071
Au	1.36	196.966 569

5 Computational Details and Theoretical Analysis

Regarding the scaling procedure, for TZP-B3LYP a factor ranging between 0.96 and 0.98 is reported in the literature [7, 8], so we used a comparison between the calculated and experimental IR spectrum to assess this scaling factor in the range 0.96 - 0.98. Such comperison gave a factor of 0.975, in agreement with the

the range reported in the literature. Once the scaling factor is obtained from IR comparison, we used the same scaling factor for VCD. The literature scaling factors for SCC-DFTB are around 1.00 suggesting a fortuitous but welcome error compensation between anharmonicity and method approximation. So we rescaled the SCC-DFTB calculation by comparison with IR experiment, for the free ligand. In this case we obtained a new scaling factor, whose value 0.99 is consistent with the literature, such value is then used for VCD of the free ligand and for both IR and VCD for the metal clusters. For the free ligand we considered all the possible conformers, and optimized them by DFT as well as SCC-DFTB calculations in order to calculate the vibrational normal modes and the VCD spectra. We also considered the Boltzmann average at room temperature, calculated by means of the DFT energies. We did not calculate the Boltzmann average by the SCC-DFTB approch since the results are very sensitive to small energy errors. So when we performed the average of the SCC-DFTB spectra we employed the Boltzmann factors calculated at the DFT level. The Boltzmann factor is calculated using energy differences between the given energy and the lowest energy conformers. Finally the factors are normalized as follows:

$$f_i = e^{-E_i/kT} / \sum_i e^{-E_i/kT}$$

In the following, we report the results obtained by studying two models for the staple structures typical of these clusters. Both structures have been extracted from the Au_{38} cluster, and the parameters of calculations are the same seen for free ligand. For the vibrational analysis we kept fixed the Au atoms, in order to simulate the normal modes of the ligand only.

5.1 Staple with 3 ligands

The calculated IR spectrum of the staple with 3 ligands has been reported in Figure 6.

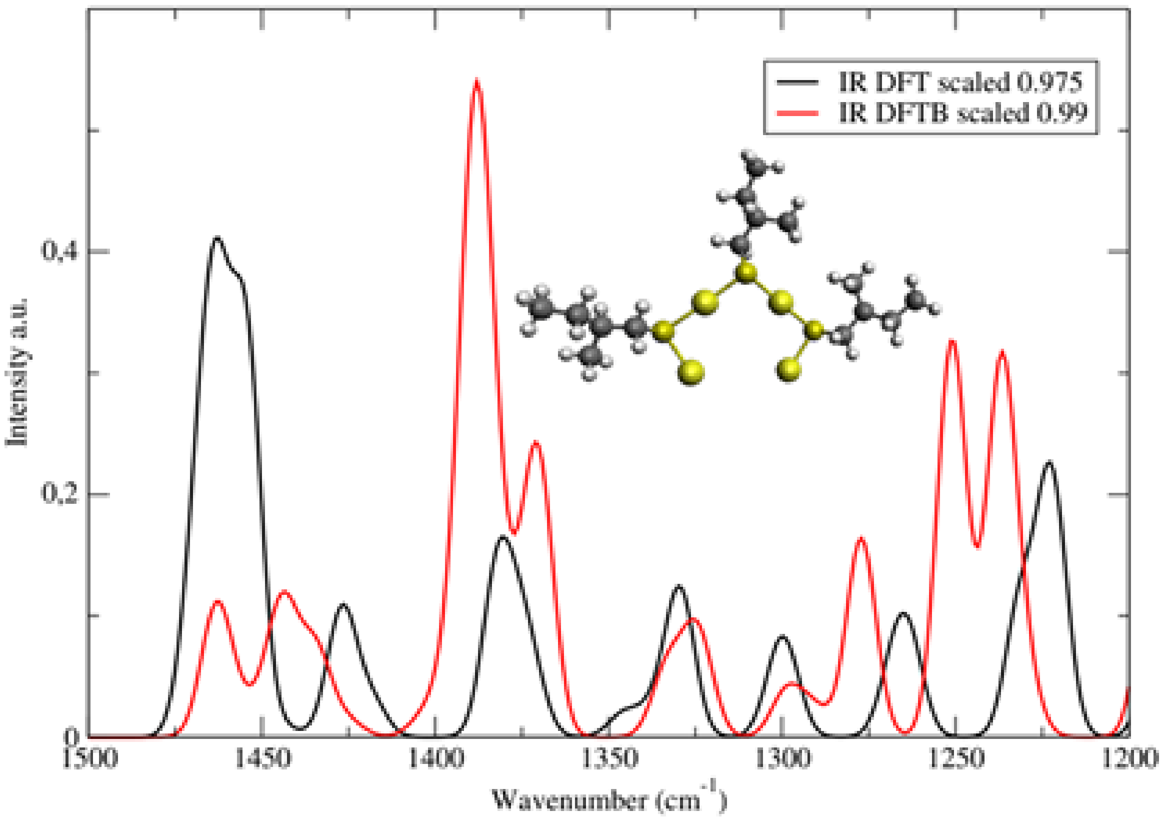

Figure 6: . Calculated (DFT and SCC-DFTB) IR spectrum of the staple with 3 ligands and 4 gold atoms.

Such staple is present in both Au_{25} and Au_{38}. The two methods give similar results of the IR although with different intensity distribution. Regarding the VCD (Figure 7) the DFT gives an exaggerated signal around 1450 cm^{-1}.

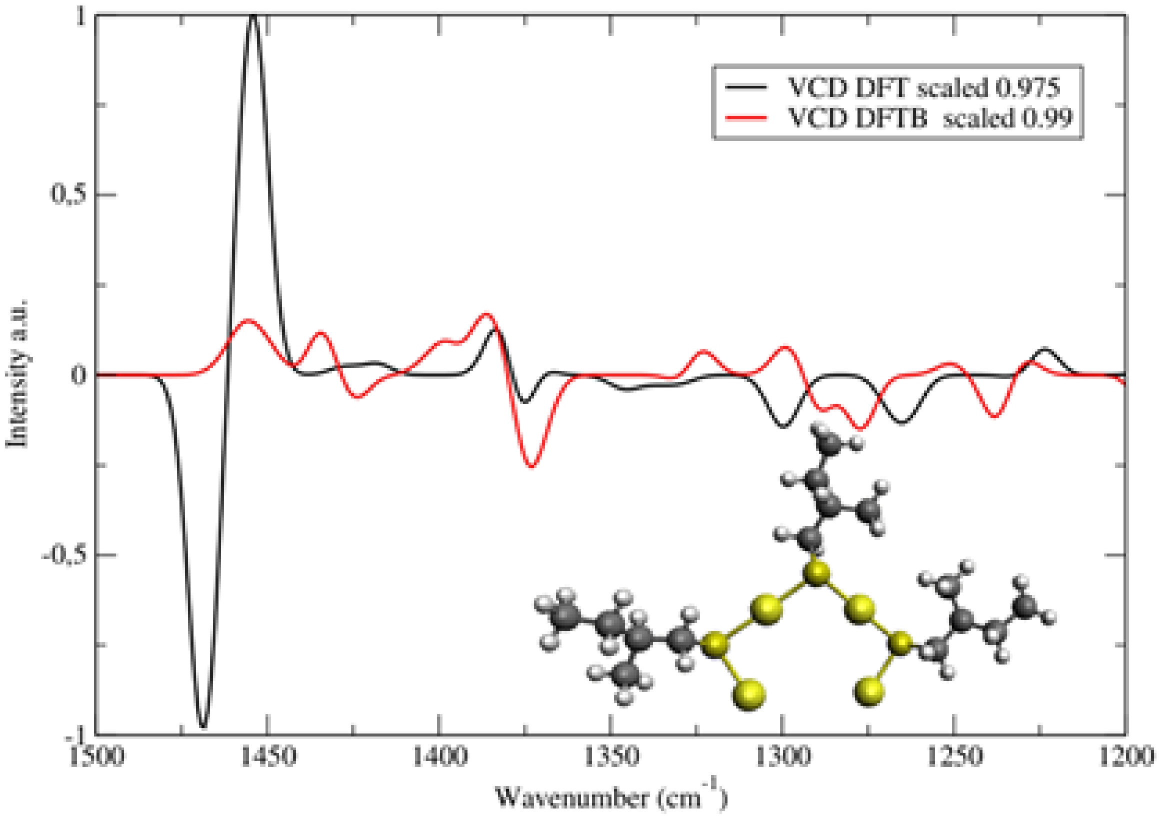

Figure 7: . Calculated (DFT and SCC-DFTB) VCD spectrum of the staple with 3 ligands and 4 gold atoms.

5.2 Staple with 2 ligands

The calculated IR spectrum of the staple with 2 ligands has been reported in Figure 8.

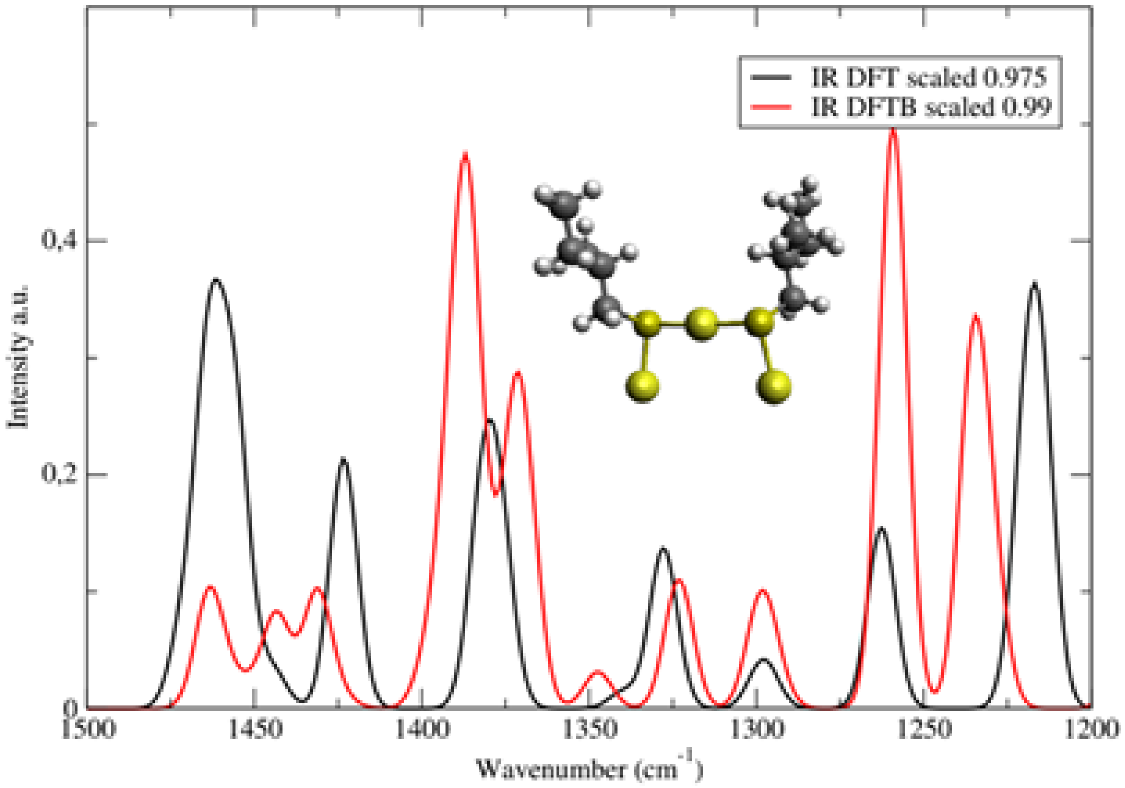

Figure 8: . Calculated (DFT and SCC-DFTB) IR spectrum of the staple with 2 ligands and 3 gold atoms.

Such staple is present in both Au_{38} and Au_{144}. The two methods give similar results of the IR although with different intensity distribution. Regarding the VCD (Figure 9) also in this case the DFT gives an exaggerated signal around 1450 cm^{-1}.

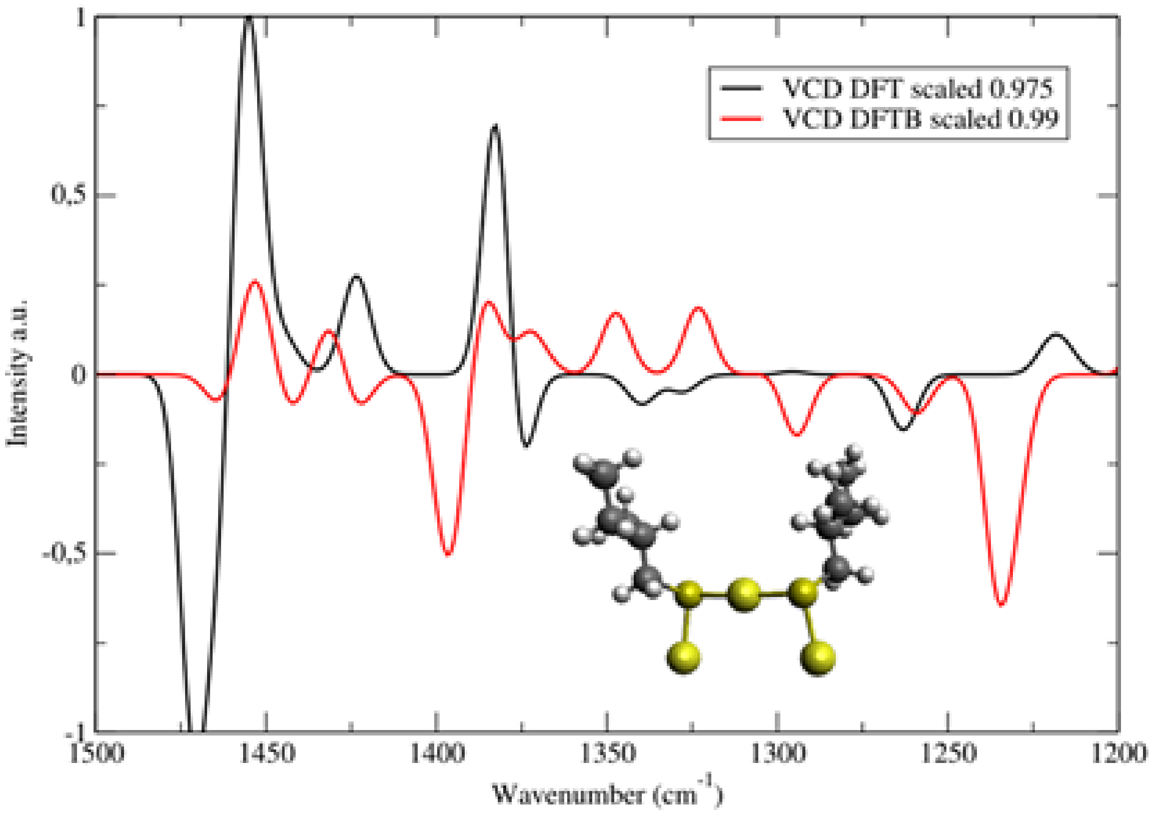

Figure 9: . Calculated (DFT and SCC-DFTB) VCD spectrum of the staple with 2 ligands and 3 gold atoms.

References

[1] Vera Truttmann, Adea Loxha, Rareş Banu, Ernst Pittenauer, Sami Malola, María Francisca Matus, Yuchen Wang, Elizabeth A. Ploetz, Günther Rupprechter, Thomas Bürgi, Hannu Häkkinen, Christine Aikens, and Noelia Barrabés. Directing intrinsic chirality in gold nanoclusters: Preferential formation of stable enantiopure clusters in high yield and experimentally unveiling the "super" chirality of au144. *ACS Nano*, 17(20):20376–20386, October 2023.

[2] Daniel Stellwagen, Andrew Weber, Gudrun Lisa Bovenkamp, Rongchao Jin, J. H. Bitter, and Challa S. S. R. Kumar. Ligand control in thiol stabilized au38 clusters. *RSC Advances*, 2(6):2276, 2012.

[3] Huifeng Qian and Rongchao Jin. Ambient synthesis of au144(sr)60 nanoclusters in methanol. *Chemistry of Materials*, 23(8):2209–2217, March 2011.

[4] Ariel F. Perez-Mellor and Riccardo Spezia. Determination of kinetic properties in unimolecular dissociation of complex systems from graph theory based analysis of an ensemble of reactive trajectories. *The Journal of Chemical Physics*, 155(12):124103, 09 2021.

[5] Ariel F. Perez Mellor, Thomas Bürgi, and Riccardo Spezia. Gas-phase reactivity of protonated oxazolone: Chemical dynamics simulations and graph theory-based analysis reveal the importance of ion–molecule complexes. *The Journal of Chemical Physics*, 162(11):114313, 03 2025.

[6] Pekka Pyykkö and Michiko Atsumi. Molecular single-bond covalent radii for elements 1–118. *Chemistry – A European Journal*, 15(1):186–197, 2009.

[7] Marco Fusè, Giovanna Longhi, Giuseppe Mazzeo, Stefano Stranges, Francesca Leonelli, Giorgia Aquila, Enrico Bodo, Bruno Brunetti, Carlo Bicchi, Cecilia Cagliero, Julien Bloino, and Sergio Abbate. Anharmonic aspects in vibrational circular dichroism spectra from 900 to 9000 cm–1 for methyloxirane and methylthiirane. *The Journal of Physical Chemistry A*, 126(38):6719–6733, 2022.

[8] I. M. Alecu, Jingjing Zheng, Yan Zhao, and Donald G. Truhlar. Computational thermochemistry: Scale factor databases and scale factors for vibrational frequencies obtained from electronic model chemistries. *Journal of Chemical Theory and Computation*, 6(9):2872–2887, 2010.
